# Fetal Exposure to Maternal Inflammation Does Not Affect Postnatal Development of Genetically-Driven Ileitis and Colitis

**DOI:** 10.1371/journal.pone.0098237

**Published:** 2014-05-21

**Authors:** Jana Hemmerling, Katharina Heller, Gabriele Hörmannsperger, Monika Bazanella, Thomas Clavel, George Kollias, Dirk Haller

**Affiliations:** 1 Chair of Nutrition and Immunology, Technische Universität München, Freising- Weihenstephan, Bavaria, Germany; 2 ZIEL - Research Center for Nutrition and Food Sciences, Freising-Weihenstephan, Bavaria, Germany; 3 Biomedical Sciences Research Centre, Institute for Immunology, Alexander Fleming, Vari, Athens, Greece; INSERM, France

## Abstract

***Background:*** Chronic inflammatory disorders have been increasing in incidence over the past decades following geographical patterns of industrialization. Fetal exposure to maternal inflammation may alter organ functions and the offspring's disease risk. We studied the development of genetically-driven ileitis and colitis in response to maternal inflammation using mouse models.

***Methods:*** Disease susceptible (*Tnf* ^ΔARE/+^ and *IL*10^−/−^) and disease-free (*Tnf* ^+/+^ and *IL*10^−/+^) offspring were raised in inflamed and non-inflamed dams. Ileal, caecal and colonic pathology was evaluated in the offspring at 8 or 12 weeks of age. Ly6G-positive cells in inflamed sections from the distal ileum and distal colon were analysed by immunofluorescence microscopy. Gene expression of pro-inflammatory cytokines was measured in whole tissue specimens by quantitative PCR. Microarray analyses were performed on laser microdissected intestinal epithelium. Caecal bacterial communities were assessed by Illumina sequencing of 16S rRNA amplicons.

***Results:*** Disease severity, the number of infiltrated neutrophils as well as *Tnf* and *Il12p40* mRNA expression were independent of maternal inflammation in the offspring of mouse models for ileitis (*Tnf* ^ΔARE/+^) and colitis (*IL*10^−/−^). Although TNF-driven maternal inflammation regulated 2,174 (wild type) and 3,345 (*Tnf* ^ΔARE/+^) genes in the fetal epithelium, prenatal gene expression patterns were completely overwritten after birth. In addition, co-housing experiments revealed no change in phylogenetic diversity of the offspring's caecal microbiota in response to maternal inflammation. This is independent of the offspring's genotype before and after the onset of tissue pathology.

***Conclusions:*** Disease risk and activity in mouse models of chronic ileitis and colitis was independent of the fetal exposure to maternal inflammation. Likewise, maternal inflammation did not alter the diversity and composition of offspring's caecal microbiota, clearly demonstrating that changes of the gene expression program in the fetal gut epithelium were not relevant for the development of chronic inflammatory disorders in the gut.

## Introduction

Lifestyle changes in industrialized countries are associated with a sharp increase in the incidence of immune-mediated chronic pathologies such as inflammatory bowel diseases (IBD), multiple sclerosis or type-1 diabetes. Th1-driven inflammatory processes are key mechanisms in the pathogenesis of these chronic pathologies[1]. IBD are spontaneously relapsing, immunologically mediated disorders of the gastrointestinal tract[2]. Although the pathogenesis of these multifactorial diseases is still not fully understood, the combination of genetic predisposition[3] and environmental factors (microbiota, diet and lifestyle)[4] drives disease development. Apart from the major impact of IBD on life quality of the affected patients, epidemiological studies link Crohn's disease (CD) activity during pregnancy to adverse outcomes such as preterm birth, spontaneous abortion and labor complications[5], [6].This is not clear in the context of ulcerative colitis.

Despite the fact that alterations of the cytokine milieu during CD-associated pregnancy lead to peri- and postnatal complications[6]–[8], the transmission of maternal inflammation to the offspring with consequences for later disease susceptibility, severity or phenotype is completely unknown. A potential impact of inflammatory processes during pregnancy on fetal organ functions was recently shown for endotoxin-induced chorioamnionitis[9]. In this context, the maturation of the fetal gut barrier was prevented [10]. Furthermore, maternal exposure to high-fat diet was recently reported to induce intestinal inflammation in fetal sheep, suggesting that even low grade maternal inflammation might affect intestinal functions and IBD susceptibility in the offspring[11]. Maternally transmitted compositional changes of the microbiota might be an important factor that contributes to this influence on disease susceptibility in the offspring [12].

In this context, we asked the question whether chronic maternal inflammation is a risk factor for postnatal disease susceptibility in the normal and genetically susceptible host. We applied sophisticated breeding systems with heterozygous *Tnf* ^ΔARE/+^ and homozygous *IL10^−/−^* mice to generate genetically-driven inflammatory disease environments *in utero* in two well-established models of chronic ileitis and colitis, enabling us to study the role of maternal inflammation on postnatal disease onset. Exemplarily, we took also advantage of the *Tnf^ΔARE/+^* model with ileitis to assess transcriptional profiling of fetal and postnatal intestinal epithelial cells (IEC) obtained by laser microdissection in order to provide high resolution of cellular specificity at this disease relevant interface. This second objective fits with the hypothesis that the pathogenesis of CD is characterized by a failure of innate immune mechanisms to recognize microbial triggers at the early stage of disease development[4]. Selective overexpression of TNF in the intestinal epithelium seems to be sufficient to trigger CD-like ileitis[13], suggesting an important role of the epithelium in the pathogenesis of chronic intestinal inflammation in *Tnf^ΔARE/+^* mice[14].

Thus, the aim of the present study was to characterize the role of genetically-driven maternal inflammation in programming the fetal epithelium towards postnatal development of intestinal inflammation.

## Materials and Methods

### Ethics Statement

Mouse experiments were performed between 2009 and 2011 in accordance to the German guidelines for animal care (Regierung von Oberbayern, Bavaria, Germany). No animal approval was obtained, because no intervention was performed with living mice. All mice were reported as mice “sacrificed for research purposes” at the Regierung von Oberbayern.

### Animals and Experimental Design

**Figure 1:**
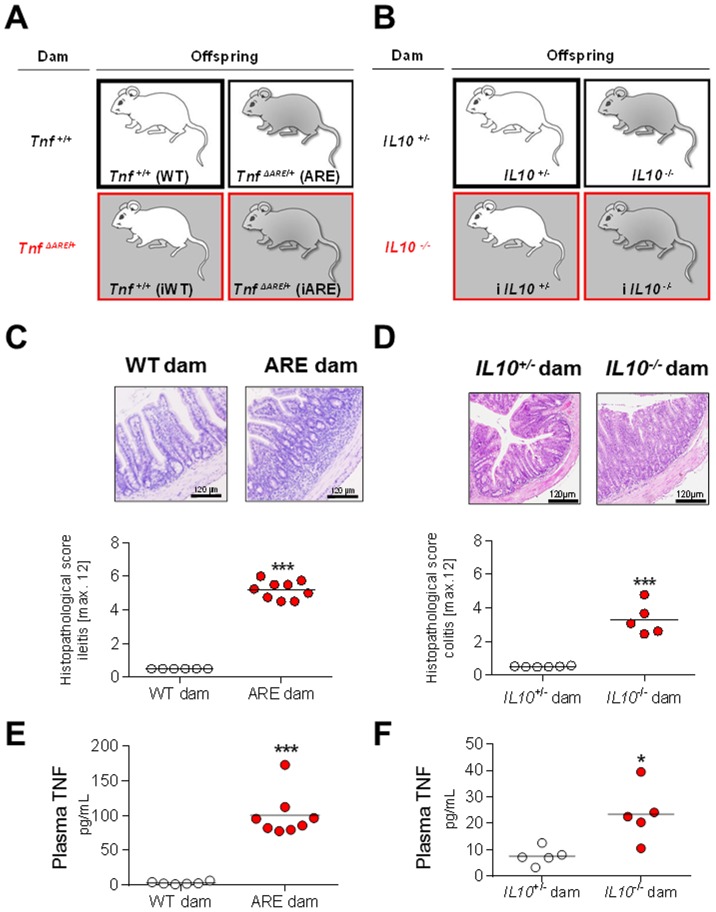
Breeding schemes and the maternal inflammatory environment. Breeding schemes of TNF- (A) and IL10-driven (B) maternal inflammation. Offspring developed under non inflamed conditions (white background: white mouse = WT or IL10+/−; grey mouse  =  ARE or IL10−/−) and under maternal inflammation (grey background: white mouse  =  iWTor iIL10+/−; grey mouse  =  iARE or iIL10−/−). (C) Representative H&E-stained transversal sections of the distal ileum and individual plots of ileitis score in WT (n = 6) and ARE (n = 8) dams. (D) Total colitis score in IL10+/− (n = 6) and IL10−/− dams (n = 8) and representative H&E-stained sections of distal colon from non-inflamed IL10+/− and inflamed IL10−/− dams. Scores [0, not inflamed, to 12, highly inflamed] were determined using tissue sections from dams sacrificed 3 weeks after giving birth. TNF in maternal plasma indicates that the inflammation is also systemically relevant in both the ileitis (E) and colitis (F) models. Individual data and means are shown; t-test, *p<0.05, ***p<0.001.

All mice were conventionally housed in groups of 3–5 mice per cage at a 12 h light/dark cycle at 24–26°C. They received fresh tap water and breeding diet (Ssniff Chow) ad libitum and were sacrificed by neck dislocation. *Tnf* ^ΔARE/+^(ARE) dams were bred with *Tnf* ^+/+^ wildtype (WT) sires (C57BL/6N genetic background) and vice versa (n = 5–10 breeding pairs), generating offspring from healthy WT dams (WT and ARE) and inflamed ARE dams (iWT and iARE) (Figure 1A) (n = 5 each). The period of conception was set from 8–12 weeks in order to avoid suffering in genetically susceptible ARE dams. Dams were sacrificed at an average age of 13 weeks +/− 5 d (WT dams) and 11 weeks +/− 1 d (ARE dams) for the prenatal time point and at 17 weeks +/− 1 d (WT dams) and 14 weeks +/− 3 d (ARE dams) for the weaning time point (3 weeks after giving birth). Offspring were sacrificed at the age of 17.5 days post conception (dpc), 3 (weaning) and 8 weeks by neck dislocation. Morphological criteria of fetuses at 17.5 dpc were evaluated according to Theiler stage 25 (TS25) based on the onset of skin wrinkling, whiskers and eyelid closure. Placentas and offspring's gut were embedded in Optimal Cutting Temperature (O.C.T.) matrix (Sakura Finetek, Torrance, USA) and stored at −80°C until laser microdissection of intestinal epithelial cells and subsequent microarray experiments.

For co-housing experiments, ARE and WT dams were kept together in one cage to generate mixed beddings from the age of 4 weeks. From week 8 on, dams were mated with WT males overnight only until pregnancy was observed. During the day, dams were co-housed again. After giving birth, beddings of ARE and WT dams and litters were exchanged to create a synchronized environment independent of the dam's genotype, so as to be able to analyse only the effect of inflammation *in utero* on shaping gut bacterial colonization. Dams and offspring were sacrificed before weaning, *i.e.*, 3 weeks after birth.

Female *IL10* ^−/−^ were bred with male *IL10* ^+/−^ (both on the 129Sv/Ev genetic background) and vice versa (n = 5–6 each), generating offspring (n =  5–15 each) from healthy *IL10* ^+/−^ dams (*IL10* ^+/−^, *IL10* ^−/−^) or inflamed *IL10* ^−/−^ dams (i*IL10* ^+/−^, i*IL10* ^−/−^) (Figure 1B). The breeding period was set between 15–21 weeks. Dams were sacrificed at weaning at an average age of 25 +/− 2 weeks (*IL10* ^−/−^ dams) and 27 +/− 4 weeks (*IL10* ^+/−^ dams). *IL10* ^−/−^ dams showing a total colitis score of at least 2 (0–12) (Figure 1 D) were considered as inflamed and the offspring was included in the analysis. Offspring were sacrificed at the age of 12 weeks by neck dislocation in order to blindly determine histological colitis scores.

### Histopathology

Scoring was performed on 10% formalin-fixed paraffin-embedded or cryo-fixed tissue for the postnatal or prenatal time point, respectively. The histological score was ascertained in a blinded fashion on H&E-stained transversal sections of the terminal ileum (WT, iWT, ARE, iARE) or of the cecum tip, proximal colon and distal colon (*IL10* ^+/−^, *IL10* ^−/−^, i*IL10* ^+/−^, *IL10* ^−/−^), resulting in a score from 0 (non-inflamed) to 12 (highly inflamed) per section as previously described[15]. The total colitis score per mouse was determined by calculating the mean of all three colonic compartments.

### Plasma Measurements of Maternal Tumor Necrosis Factor

Plasma TNF was measured using Mouse TNF alpha ELISA Ready –SET-Go! ELISA, according to the manufacturer's instructions (eBioscience, San Diego, USA). A volume of 100 µl total plasma was incubated on a pre-coated plate for 2 h at RT (room temperature), followed by incubation of anti-TNF detection antibody linked with Avidin/Biotin. HRP-conjugated antibody was incubated for 30 min and substrate conversion was stopped after 15 min with 2N H_2_SO_4_. The product absorbance of standard dilutions and plasma sample was measured at 405 nm to the reference wavelength of 570 nm. Quantification was performed using the linear equation of the standard dilutions.

### Immunofluorescence Staining of Ly6G and REG3B in the Intestine

Transversal sections (5 µm thick) were cut from either formalin-fixed paraffin embedded (FFPE) or from frozen distal ileum or colon and transferred onto Superfrost Plus slides (Thermo Scientific, Braunschweig, Germany). After deparaffinization of FFPE tissue (Leica ST5020 Multistainer system), antigen demasking was performed by boiling in 1× sodium citrate buffer (pH 6, 900 W, 23 min). After cool down to RT, slides were washed 3 times in dH2O for 5 min, followed by 5 min in PBS. Frozen sections were equilibrated to room temperature (30 min), fixed in ice cold acetone (−20°C) for 10 min, followed by 30 min air drying at RT and 3 times 5 min washing in PBS. Sections were blocked with 50 µl blocking buffer raised against the species of the secondary antibody for 60 min at RT in a humidified chamber. Primary antibody against Ly6G (rat-anti-Ly6G, BD Pharmingen, 1∶500) was incubated overnight at 4°C. Primary antibody against REG3B (sheep- anti-REG3B, R&D Systems, 1∶100 dilution) was incubated at RT for 1–3 hours in a humidified chamber. Fluorochrome-conjugated secondary antibodies, namely goat anti-rat IgG (H+L) Alexa Fluor 546 (Invitrogen) or donkey anti-sheep IgG (H+L) DyLight 488 (Jackson ImmunoResearch), were diluted 1∶200 and incubated for 1 h at RT. Nuclei were counterstained using DAPI (1∶2000) in secondary antibody solution. Sections were visualized using a confocal microscope (Olympus Fluoview 1000 using the FV10-ASW software). The amount of Ly6G positive cells per area was counted using the Volocity 5.51 software (Perkin Elmer) defining the lamina propria as region of interest. For each individual mouse, 3 microscopic fields at a 600-fold magnification were quantified for mean Ly6G-positive cells per mm^2^. Immunofluorescence intensity of REG3B was quantified with Volocity Demo 5.5 software (Perkin Elmer) defining epithelial cells as region of interest. For each individual mouse 3 different areas were quantified as mean intensity of the fluorescence signal per µm^2^.

### Gene Expression Analysis of Whole Gut Tissue and Laser Microdissected IEC

RNA from cryosections of distal ileum (3 sections of 10 µm each) and colonic swiss rolls (2 sections of 10 µm each) was isolated using the RNA isolation kit according to the manufacturer's instructions (Macherey & Nagel). Reverse transcription was performed using SuperScript III Reverse Transcriptase (Invitrogen). RNA of laser microdissected IEC (10 ng) was preamplified with the QuantiTect Whole Transcriptome Kit (Oiagen, Hilden, Germany) according to the manufacturer's instructions. Gene-specific nucleotide sequences and accession numbers were obtained from the National Center for Biotechnology Information (NCBI) website (http://www.ncbi.nlm.nih.gov/gene). Primer pairs (Table 1) were designed using the Universal ProbeLibrary (UPL) design center (Roche Diagnostics, Mannheim, Germany). Quantitative real-time PCR was performed on 10 ng cDNA using the LightCycler 480 System (Roche Diagnostics, Mannheim, Germany). Crossing points (Cp) were determined using the second derivative maximum method by the LightCycler 480 software release 1.5.0. Data were normalized to the Cp mean of reference genes (*Gapdh* for whole gut tissue and *18s*, *Rpl13a* and *Gapdh* for pre-amplified cDNA of IEC) and expressed as 2^−ΔCt^ values in order to compare expression levels among all groups.

**Table 1:** Primer sequences and UPL probe IDs for qPCR analysis.

Gene	Forward primer	Reverse primer	Probe	Amplicon
*Gapdh*	5′-tcc act cat ggc aaa ttc aa	5′-ttt gat gtt agt ggg gtc tcg	#9	108 nt
*Rpl13a*	5′-atc cct cca ccc tat gac aa	5′-gcc cca ggt aag caa act t	#108	97 nt
*18s*	5′-aaa tca gtt atg gtt cct ttg gtc	5′-gct cta gaa tta cca cag tta tcc aa	#55	67 nt
*C3*	5′-acc tta cct cgg caa gtt tct	5′-ttg tag agc tgc tgg tca gg	#76	75 nt
*Il12p40*	5′-atc gtt ttg ctg gtg tct cc	5′-gga gtc cag tcc acc tct aca	#78	80 nt
*Tnf*	5′-tgc cta tgt ctc agc ctc ttc	5′-gag gcc att tgg gaa ctt ct	#49	117 nt

Housekeeping genes are underlined.

### Laser Microdissection (LMD) Microscopy of Intestinal Epithelial Cells

Ileal cryo-sections (Microm, Walldorf, Germany) were generated at −20°C. PET frame slides (MicroDissect, Herborn, Germany) were treated with RNase ZAP (Sigma-Aldrich, Steinheim, Germany) before use and dried at RT. Transversal sections (10 µm) were mounted on slides, air-dried and stored at −80°C for short periods of time (<7 d) until use. Each slide was stained directly before LMD microscopy. Briefly, after equilibration to RT (2 min), slides were fixed for 1 min with 70%(v/v) EtOH, rinsed with Diethylpyrocarbonate (DEPC) water for 30 sec, stained with Harris hematoxylin for 1 min and rinsed with DEPC water for 30 sec. After bluing with 0.1% (v/v) NH_4_OH for 30 sec, slides were counterstained with 2.5% Eosin for 2 min. Finally, sections were dehydrated in ascending EtOH series (96%, 100%) for 30 sec each and air dried at RT for 5 min. Ileal IEC were cut at a magnification of 630× using the UV laser-cutting system LMD 6000 and the Leica Application Suite software (Leica, Wetzlar, Germany). Lysis buffer (100 µl) supplied in the AllPrep DNA/RNA Micro Kit (Qiagen, Hilden, Germany) was added to epithelial pieces directly after dissection. Samples were kept frozen at −80°C until DNA/RNA isolation. In total, a mean area of 1.330±0.024×10^6^ µm^2^ IECs was collected per sample (Figure S1).

### RNA Isolation and Quality Control

Total RNA was isolated using the column-based AllPrep DNA/RNA Micro Kit (Qiagen, Hilden, Germany). RNA concentration was measured using the Quant-iT RiboGreen RNA Assay Kit (Invitrogen, Eugene, USA). RNA integrity was determined using the RNA 6000 Pico Kit and the Bioanalyzer 2100 (Agilent, Waldbronn, Germany). An amount of 50 ng total RNA (3 µl sample volume adjusted by vacuum centrifugation) was used for microarray analysis.

### Microarray-based Gene Expression Analysis

Microarray analysis was performed with ileal IEC from 17.5 *dpc* and 8-week-old mice (n = 20 in total). All samples from one time point were run together. RNA preparation, reverse-transcription, amplification and biotin labeling were performed using the GeneChip 3′ IVT Express kit (Affymetrix, Santa Clara, USA). Hybridization, washing and staining were performed using the GeneChip Hybridization, Wash and Stain (Affymetrix) and the GeneChip Fluidics Station 450 (Affymetrix). Labeled RNA samples were hybridized to customized murine genome NuGO_Mm1a520177 microarrays containing 23,865 probe sets (covering 15,313 genes, Affymetrix). Gene chips were visually inspected for irregularities and scanned with GeneChip Scanner 3000. Data were analyzed using the Affymetrix GCOS Manager software and the R- and Bioconductor-based MADMAX interface (Management and Analysis Database for Multi-platform microArray eXperiments, https://madmax.bioinformatics.nl), including R version 2.11.1, Bioconductor version 2.6 and AnnotationDbi version 1.10.2. Data were normalized using the gc Robust Multichip Average (slow) algorithm. Probe sets were annotated to the transcripts with Custom Chip Definition files version 13.0.0. (nugomm1a520177mmentrezg.cdf). No previous filtering was applied to the datasets. MADMAX calculated Fold Changes (FCs), *p*-values, FDR-values and q-values to the study groups with the LIMMA procedure. The LIMMA (log2 based) FCs were calculated comparing iWT, ARE and iARE groups to the WT control group (n = 5 each). Genes were considered to be significantly regulated according to the raw *p*-value of LIMMA with *p*<0.05 and a threshold FC of ±1.5. Heatmaps were generated using the MultiExperiment Viewer (TigrMEV) software. Gene Ontology (GO) terms were computed using the GeneRanker program (Genomatix, München, Germany). Overrepresentation of biological terms were calculated and listed in the output together with respective *p*-values.

### Illumina Sequencing of 16S rRNA Gene Amplicons from Caecal Contents

Bacterial DNA was obtained after bead beating and ethanol precipitation [16] from directly frozen caecal contents (co-housing experiment) or from ceacal contents embedded in O.C.T. Amplicons of the V4 region of 16S rRNA genes were obtained after 25 PCR cycles as described previously[17]. They were sequenced in paired-end modus (PE200) using the MiSeq system (Illumina Inc., San Diego, USA). Sequences were analyzed using in-house developed pipelines partly based on UPARSE [18], the open source software package QIIME[19] and the Ribosomal Database Project[20]. Sequences were filtered at a base call accuracy of 99%. Sequences containing any ambiguous nucleotide (N character) were discarded. The presence of chimeras was checked after dereplication using UCHIME [21]. Operational taxonomic units (OTUs) were picked at a threshold of 97%. Only those OTUs occurring in at least one sample at abundances >0.5% total sequences were included in the analysis. Sequence proportions of bacterial taxa were analyzed for significant differences using F-Test followed by Benjamini-Hochberg correction for multiple testing in the R programing environment (2008, ISBN 3-900051-07-0).

### Statistics

Statistical analyses were performed with SigmaPlot 11.0 using unpaired t-test, Kruskal-Wallis test followed by Dunn's multiple comparison or ANOVA followed by pairwise comparisons testing (Holm-Sidak test). Data were expressed as mean ± SD. Differences between groups were considered significantly if *p*-values were <0.05 (*), <0.01 (**), <0.001 (***). Graphics were created using GraphPad Prism version 5.00 (GraphPad software, San Diego, USA).

## Results

### Maternal Inflammation does not Influence Postnatal Development of Intestinal Inflammation

**Figure 2:**
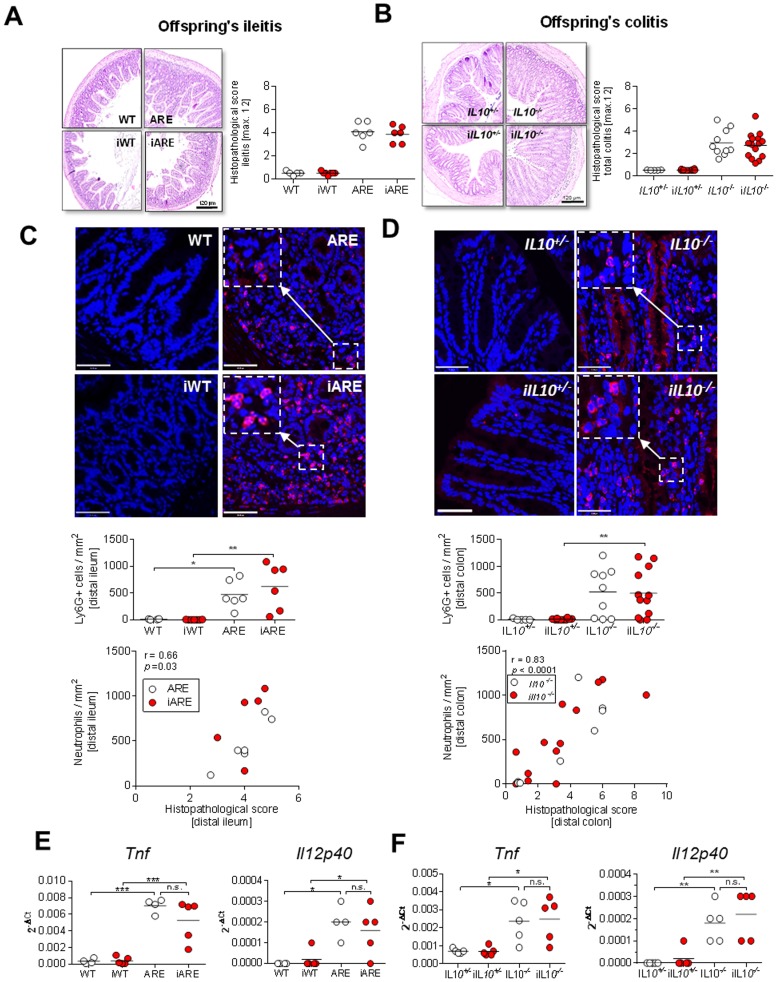
Postnatal tissue inflammation in *Tnf^ΔARE/+^* and *IL10^−/−^* mice is not affected by maternal inflammation. (A) Ileitis scores from WT, iWT, ARE and iARE (each n = 5–7) offspring sacrificed at 8 weeks of age with representative H&E stained sections of the distal ileum. (B) Total colitis scores from *IL10* ^+/−^, *iIL10* ^+/−^, *IL10* ^−/−^ and i*IL10* ^−/−^ offspring at 12 weeks of age (n = 5–15) with representative H&E-stained sections of the distal colon. Individual data and means are shown, *p<0.05, *** p<0.001 Kruskal-Wallis Test with Dunn's multiple comparisons. (C+D) Representative microscopic pictures (600× magnification) of confocal laser microscopy for Ly6G-immunofluorescence (red) from distal ileum in WT, iWT, ARE and iARE offspring and from distal colon in *IL10^+/−^*, i*IL10^+/−^, IL10^−/−^* and i*IL10^−/−^* offspring. Nuclei are counterstained with DAPI (blue). Three pictures per mouse were analyzed. Lamina propria and submucosa were defined as regions of interest. The numbers of Ly6G-positive cells per mm^2^ from all 3 pictures per mouse were counted. Individual data and means are shown (Two-Way ANOVA, *p<0.05, **p<0.01). Correlation analysis in *Tnf^ΔARE/+^* and *IL10^−/−^* offspring indicated strong associations between histopathological scores and infiltration of Ly6G-positive neutrophils. (E+F) Whole tissue specimens were analyzed for *Tnf* and *Il12p40* gene expression in offspring from ileitis and colitis mouse models as described in the method section. RNA was isolated from distal ileal cryosections (3×10 µm) of WT, iWT, ARE and iARE offspring and from colonic swiss rolls of *IL10^+/−^*, i*IL10^+/−^*, *IL10^−/−^* and i*IL10^−/−^* offspring (n = 5 each). Single values and means are indicated as 2^−ΔCt^. Two Way ANOVA, *p<0.05, **p<0.001, ***p<0.0001.

Conventionally raised *Tnf* ^ΔARE/+^ (ARE) and *IL10* ^−/−^ mice were used to study the impact of maternal inflammation on the intestine in healthy and genetically susceptible offspring. As expected, all ARE dams were affected by moderate inflammation in the distal ileum (score 5.2 ± 0.5) (Figure 1C). *IL10* ^−/−^ dams showed a more heterogeneous inflammation in the large intestine and an inflammation score of 2 was set as minimal value to discriminate inflamed dams, resulting in a mean inflammatory score of 3.3 ± 0.8 for the dams that were included in the study (Figure 1D). The local inflammation in the intestine of dams was reflected systemically by significantly elevated TNF levels in the plasma of both inflamed *Tnf^ΔARE/+^* (2.9 ± 1.9 pg/mL vs. 100.0± 31.6 pg/mL) and *IL10* ^−/−^ mice (7.6 ± 3.3 pg/mL vs. 23.5 ± 10.4 pg/mL) (Figures 1E and F). The abundance of plasma TNF significantly correlated with the inflammation grade in the intestine (data not shown). The intestinal inflammation of healthy and genetically susceptible offspring was found to be unaffected by maternal inflammation in both models at the age of 8 weeks (ileitis) and 12 weeks (colitis) (Figure 2A and B). Both, ARE and iARE mice developed moderate ileitis with comparable histological scores (4.4 ± 0.9 and 4.0 ± 0.7). Likewise, *IL10* ^−/−^ and i*IL10* ^−/−^ offspring showed similar histological grades of colitis (3.0 ± 1.1 and 2.7 ± 1.1). There was no correlation between maternal and offspring's intestinal inflammation (data not shown). Additionally, the number of infiltrated Ly6G-positive cells into inflamed tissue of the distal ileum of *Tnf^ΔARE/+^* offspring or the distal colon of *IL10* ^−/−^ offspring was unaffected by maternal inflammation (ARE vs. iARE: 473 ± 262 cells/mm^2^ vs. 621 ± 434 cells/mm^2^; *IL10* ^−/−^ vs. i*IL10* ^−/−^: 521 ± 454 cells/mm^2^ vs.497 ± 441 cells/mm^2^) (Figures 2C and D). The variation of neutrophil numbers in the distal colon of *IL10* ^−/−^ was strongly associated (r = 0.82, p<0.0001) with histological scores in the distal colon, which showed high heterogeneity (total colitis score from 0.6 to 8.8). The fact that histological scores were not affected by maternal inflammation was supported by unchanged mRNA expression of pro-inflammatory cytokines in whole gut sections from the distal ileum (*Tnf* ^ΔARE/+^) and colonic swiss rolls (*IL10* ^−/−^) between the different offspring groups (Figures 2E and F). In other words, *Tnf* and *Il12p40* mRNA levels were not altered between ARE and iARE or *IL10* ^−/−^ and i*IL10* ^−/−^, but were significantly increased between WT and ARE or *IL10* ^+/−^ and *IL10* ^−/−^ offspring. All these data consistently show that maternal inflammation does not impact genetically-driven disease phenotypes in both mouse models of intestinal inflammation. In addition, these results raised the question of whether there is any early transcriptional programming effect on the offspring's epithelium that is induced by maternal inflammation.

### Transcriptional Fingerprints of the Fetal Epithelium in Response to Maternal Inflammation do not Persist in Grownup Mice

**Figure 3:**
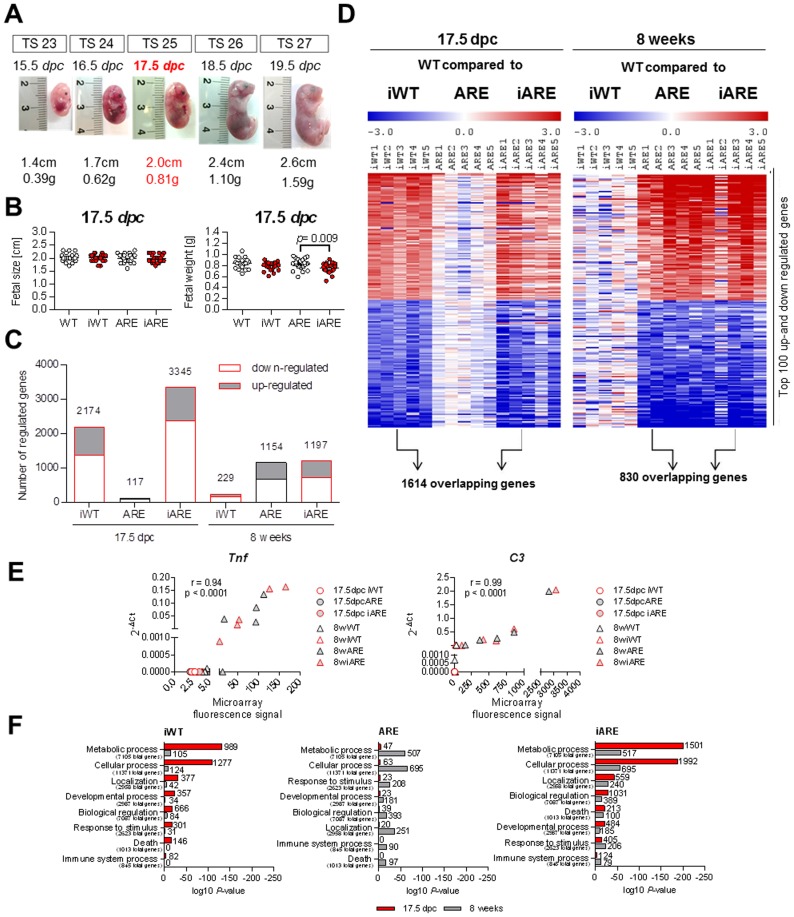
Fetal gene expression profiles in the ileal epithelium are overwritten by postnatal inflammation. (A) Randomly selected fetuses of the last 5 Theiler stages (TS) (15–19 *dpc*). (B) 17.5 *dpc* fetus genotypes plotted against fetal size and weight (total of 98 fetuses); Two-Way ANOVA followed by Holm-Sidak test, *p<0.05, ***p<0.001. (C) Number of regulated genes in iWT, ARE and iARE mice at 17.5 *dpc* and 8 weeks of age when compared to WT considering a threshold fold change of ± 1.5, *p*<0.05 (each group and time point n = 5). (D) Heat map of top-100 up- and down-regulated genes in iWT, ARE and iARE mice plotted as signal log ratios from −3 to 3. Two distinct gene clusters are shown between maternal (fetal iWT and iARE) and postnatal inflammation (ARE and iARE). Data are based on corresponding WT control mice according to MADMAX statistical analysis. (E) Microarray validation of the *Tnf* and *C3* genes by plotting individual microarray fluorescence intensities against 2^−ΔCt^ values obtained by qPCR from both prenatal and postnatal mice. Correlation coefficient (r) and significance levels are indicated in the respective plots. (F) Significantly overrepresented GO_BP of prenatal (red bars) and postnatal (grey bars) iWT, ARE and iARE mice. Numbers below the GO terms indicate total genes for the particular process. Numbers next to the bars indicate observed numbers of significantly regulated genes belonging to the respective process.

Selective overexpression of TNF in the intestinal epithelium is sufficient to trigger CD-like ileitis[13], suggesting an important role of the epithelium in the pathogenesis of chronic intestinal inflammation in *Tnf^ΔARE/+^* mice. Furthermore, TNF plays a pivotal role in the development of CD, which has been effectively treated with anti-TNF agents[22]. To further characterize the role of maternal inflammation on the offspring's intestinal homeostasis, we focused on the impact of maternal inflammation on IEC. Since *IL10^−/−^* dams and offspring showed variable degrees of disease severity along the large intestine, we focused on offspring from *Tnf* ^ΔARE/+^ and *Tnf* ^+/+^ mice to perform this more detailed cell-specific analysis. Therefore, we measured gene expression profiles in IEC at pre- (17.5 *dpc*) and postnatal (8 weeks) time points using laser dissected ileal epithelium. Randomly selected fetuses of different stages (15–19 *dpc*) are shown in Figure 3A. At 17.5 *dpc*, fetuses had comparable sizes, but body weight was decreased in iARE compared to ARE fetuses (Figure 3B). Equivalent surface areas of laser microdissected IEC were collected in all groups (Figure S1A). RNA integrity numbers (RIN) were 5.0 ± 0.8 and 5.5 ± 1.2 for fetal and postnatal RNA, respectively.

Gene expression profiles showed two distinct patterns according to maternal and postnatal inflammation (GEO data base accession number GSE44433). In fetal IEC, gene expression was highly influenced by maternal inflammation with 2,174 and 3,345 significantly regulated genes in iWT and iARE progeny, respectively (Figure 3C). Heatmap comparisons showed similar patterns in iWT and iARE, indicating that similar genes were regulated under conditions of maternal inflammation (1,614 common genes) (Figure 3D, Table 2). The 5 most up- and down-regulated genes at pre- and postnatal time points are shown in Table 3. *Reg3b* (*regenerating islet-derived 3 beta*) and *Fabp6* (*fatty acid binding protein 6*) were identified among the top-regulated genes under maternal inflammation. The fetal ARE genotype was apparently not relevant at this early gut developmental stage (only 117 regulated genes). In contrast to the fetal stage, gene expression profiles in postnatal IEC were highly influenced by the ARE genotype with 1,154 and 1,197 significantly regulated genes under ARE and iARE conditions, respectively (Figure 3A). Only 229 genes were significantly regulated in iWT compared to WT mice, indicating that the postnatal environment and the disease susceptible genotype of the offspring almost completely overwrote the gene expression program in the fetal gut. Heatmap analysis confirmed these different patterns and showed that postnatal tissue inflammation (ARE and iARE) most strikingly triggered *C3* and *S100a8* gene expression. The majority of regulated genes were shared according to postnatal tissue inflammation (830 commonly regulated genes between ARE and iARE mice).

**Table 2:** Top 10 up- and down-regulated overlapping genes between prenatal WT/iWT and WT/iARE gene expression patterns (effect of maternal inflammation) and between postnatal WT/ARE and WT/iARE gene expression patterns (effect of offspring genotype/disease).

Overlapping genes between WT/iWTand WT/iARE at 17.5 dpc				Overlapping genes between WT/ARE and WT/iARE at 8 weeks			
ID	Gene	FC iWT	FC iARE	ID	Gene	FC ARE	FC iARE
18489	*Reg3b*	22.45	15.53	12266	*C3*	39.04	37.53
16204	*Fabp6*	15.61	30.29	20201	*S100a8*	38.08	21.38
58861	*Cysltr1*	6.69	4.20	17105	*Lyz2*	25.47	21.82
319636	*Fsd1l*	6.66	8.17	68891	*Cd177*	18.34	16.52
17967	*Ncam1*	5.58	4.72	17388	*Mmp15*	16.11	16.31
12843	*Col1a2*	5.29	3.04	70045	*2610528A11Rik*	15.46	14.98
12660	*Chka*	5.17	5.74	20202	*S100a9*	15.46	8.83
12552	*Cdh11*	4.82	2.04	22418	*Wnt5a*	14.78	15.00
77700	*9130208D14Rik*	4.68	3.47	14990	*H2-M2*	14.42	17.06
19876	*Robo1*	4.54	4.22	13419	*Dnase1*	11.42	16.73
208677	*Creb3l3*	−4.44	−1.66	105387	*Akr1c14*	−8.71	−12.00
18605	*Enpp1*	−5.13	−5.35	70564	*5730469M10Rik*	−8.82	−5.89
68979	*Nol11*	−5.54	−8.16	13487	*Slc26a3*	−8.96	−7.01
381259	*Als2cr4*	−5.62	−4.79	14344	*Fut2*	−9.18	−8.35
667373	*Gm14446*	−5.81	−2.39	109731	*Maob*	−9.59	−8.39
100647	*Upk3b*	−6.01	−7.90	17161	*Maoa*	−12.93	−15.73
66350	*Pla2g12a*	−7.53	−6.07	170752	*Bco2*	−16.1	−19.65
19336	*Rab24*	−8.62	−6.47	432720	*Akr1c19*	−17.17	−10.11
107272	*Psat1*	−9.03	−11.71	12116	*Bhmt*	−22.04	−23.73
12696	*Cirbp*	−10.18	−7.56	16173	*Il18*	−25.04	−24.48

FC = log 2 based fold change, *p*<0.05.

**Table 3:** Top 5 up- and down-regulated genes in iWT, ARE and iARE mice pre- and postnatally.

5 most up- and down- regulated genes								
17.5 dpc					8 weeks			
	ID	Gene	Description	FC	ID	Gene	Description	FC
iWT	18489	Reg3b	regenerating islet-derived 3 beta	22.45	14170	Fgf15	fibroblast growth factor 15	4.50
	16204	Fabp6	fatty acid binding protein 6, ileal (gastrotropin)	15.61	56485	Slc2a5	solute carrier family 2, member 5	4.15
	58861	Cysltr1	cysteinyl leukotriene receptor 1	6.69	224093	Fam43a	family with sequence similarity 43, member A	2.87
	319636	Fsd1l	fibronectin type III and SPRY domain containing 1-like	6.66	64452	Slc5a4a	solute carrier family 5, member 4a	2.73
	17967	Ncam1	neural cell adhesion molecule 1	5.58	17388	Mmp15	matrix metallopeptidase 15	2.48
	12696	Cirbp	cold inducible RNA binding protein	−10.18	20753	Sprr1a	small proline-rich protein 1A	−3.88
	107272	Psat1	phosphoserine aminotransferase 1	−9.03	14583	Gfpt1	glutamine fructose-6-phosphate transaminase 1	−3.03
	19336	Rab24	RAB24, member RAS oncogene family	−8.62	625599	Gml	GPI anchored molecule like protein	−2.97
	66350	Pla2g12a	phospholipase A2, group XIIA	−7.53	71578	Sval1	seminal vesicle antigen-like 1	−2.80
	14607	Gip	gastric inhibitory polypeptide	−6.38	20210	Saa3	serum amyloid A 3	−2.73
ARE	18489	Reg3b	regenerating islet-derived 3 beta	3.73	12266	C3	complement component 3	39.04
	69814	Prss32	protease, serine, 32	3.41	20201	S100a8	S100 calcium binding protein A8 (calgranulin A)	38.08
	16204	Fabp6	fatty acid binding protein 6, ileal (gastrotropin)	3.23	17105	Lyz2	lysozyme 2	25.47
	19662	Rbp4	retinol binding protein 4, plasma	2.37	68891	Cd177	CD177 antigen	18.34
	56312	Nupr1	nuclear protein 1	2.02	17388	Mmp15	matrix metallopeptidase 15	16.11
	259301	Leap2	liver-expressed antimicrobial peptide 2	−3.60	16173	Il18	interleukin 18	−25.04
	56012	Pgam2	phosphoglycerate mutase 2	−2.69	12116	Bhmt	betaine-homocysteine methyltransferase	−22.04
	106861	Abhd3	abhydrolase domain containing 3	−2.66	432720	Akr1c19	aldo-keto reductase family 1, member C19	−17.17
	23958	Nr2e3	nuclear receptor subfamily 2, group E, member 3	−2.51	170752	Bco2	beta-carotene oxygenase 2	−16.10
	14058	F10	coagulation factor X	−2.28	17161	Maoa	monoamine oxidase A	−12.93
iARE	16204	Fabp6	fatty acid binding protein 6, ileal (gastrotropin)	30.29	12266	C3	complement component 3	37.53
	14963	H2-Bl	histocompatibility 2, blastocyst	26.14	17105	Lyz2	lysozyme 2	21.82
	18489	Reg3b	regenerating islet-derived 3 beta	15.53	20201	S100a8	S100 calcium binding protein A8 (calgranulin A)	21.38
	67092	Gatm	glycine amidinotransferase	12.39	14990	H2-M2	histocompatibility 2, M region locus 2	17.06
	319636	Fsd1l	fibronectin type III and SPRY domain containing 1-like	8.17	13419	Dnase1	deoxyribonuclease I	16.73
	545369	Gm5835	predicted gene 5835	−12.81	16173	Il18	interleukin 18	−24.48
	107272	Psat1	phosphoserine aminotransferase 1	−11.71	12116	Bhmt	betaine-homocysteine methyltransferase	−23.73
	665146	Gm7517	predicted gene 7517	−11.21	170752	Bco2	beta-carotene oxygenase 2	−19.65
	68979	Nol11	nucleolar protein 11	−8.16	17161	Maoa	monoamine oxidase A	−15.73
	12466	Cct6a	chaperonin containing Tcp1, subunit 6a (zeta)	−7.92	105387	Akr1c14	aldo-keto reductase family 1, member C14	−12.00

Fold changes refer to WT control mice according to MADMAX statistical analysis (n = 5 per group, *p*<0.05; FC = log 2 based fold change).

The two distinct gene expression patterns (pre- and postnatal) were reflected as well in the Gene Ontology (GO) terms for ‘biological processes’ (GO_BP) (Figure 3F). ‘Metabolic’ (GO:0008152) and ‘cellular processes’ (GO:0009987) were predominantly overrepresented according to maternal inflammation in the prenatal state and according to postnatal inflammation in adult mice, respectively. For example, among ‘metabolic processes’, 989 (iWT) and 1,501 (iARE) genes were significantly regulated in the fetal stage. *Fabp6* was highly up-regulated due to maternal inflammation and is part of both ‘metabolic’ and ‘cellular processes’. In the absence of maternal inflammation, fetal IEC from ARE mice were characterized by only 47 significant genes involved in ‘metabolic processes’ (Figure 3F, red bars). In contrast, the transcriptional profile of postnatal IEC revealed a lower significance of association (log10 *p*-value) for ‘biological processes’, highly influenced by postnatal but not maternal inflammation. In ARE mice, 507 and 695 genes were significantly regulated in ‘metabolic’ and ‘cellular processes’, respectively, whereas in iWT mice only 105 and 124 genes were responsible for overrepresentation (Figure 3F, grey bars). At this postnatal stage, *C3* encoding complement component 3 had the strongest impact on ‘metabolic’ and ‘cellular processes’ in ARE and iARE groups, but not in iWT mice.

### Microarray Validation of *Tnf* and *C3* mRNA Expression by qPCR

In order to validate the microarray data, we performed targeted gene expression analysis of two candidate genes via qPCR. Our first target was *Tnf* mRNA, which is stabilized in the genetically engineered *Tnf^ΔARE/+^* mouse model and therefore served as control. We observed a very high correlation between microarray fluorescence data and 2^−ΔCt^ values (Figure 3E). In the prenatal stage, *Tnf* was not expressed and not regulated in both microarrays and qPCR. At 8 weeks of age, when the inflammation in the offspring was fully established, *Tnf* was highly up-regulated in *Tnf^ΔARE/+^* mice in both datasets. These data mirror the increased *Tnf* transcript levels in whole distal ileal tissues from *Tnf^ΔARE/+^* offspring seen in Figure 2E. We further analyzed *complement component C3* (*C3*), which was identified by microarray analysis as the top up-regulated gene between WT and ARE offspring (FC 39.04). We again observed a high correlation between the microarray fluorescence intensities and qPCR results (Figure 3E). *Reg3b* was identified as the strongest induced gene under maternal inflammation at 17.5 *dpc*, albeit at low gene expression levels (GEO data base accession number GSE44433). In essence, the success of microarray validation is fragile as many factors can influence both methodologies [23]. For instance, low array spot intensities for *Reg3b* ranged from ∼ 10–300 (prenatal stage) and might have resulted in a discrepancy of results indicated by a low correlation between microarray fluorescence intensities and 2^−ΔCt^ values (r = 0.51, p =  0.0007). Therefore, we investigated the intestinal expression of REG3B at the level of proteins.

### Maternal Inflammation Influences REG3B Protein Expression in Adult WT Offspring, but has no Influence on the Intestinal Inflammation in *Tnf^ΔARE/+^* Offspring

**Figure 4:**
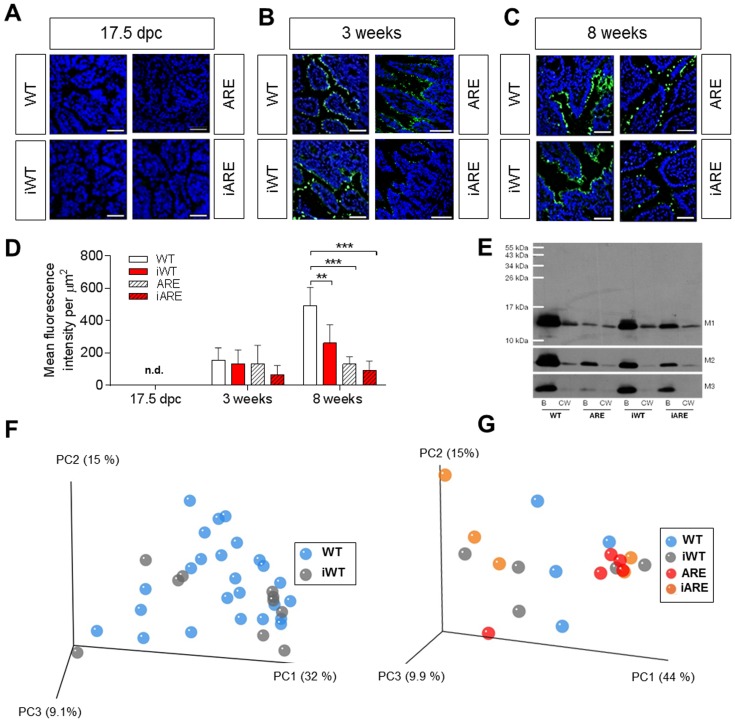
Pre- and postnatal REG3B protein expression in the distal ileum and offspring's caecal bacterial diversity are unaffected by maternal inflammation. (A–C) Immunofluorescence analysis overtime (17.5 dpc, 3 and 8 weeks) of REG3B (green) from distal ileal sections of WT, iWT, ARE, iARE mice. Nuclei were counterstained with DAPI (blue). (D) Data represent mean fluorescence intensity of REG3B signal per µm^2^ ±SD from 5 mice per group (3 regions per mouse were evaluated). Significant differences in comparison to WT mice were assessed by Two-Way ANOVA followed by Holm-Sidak method; **p<0.01, ***p<0.001, n.d. = not detectable. (E) Western blot analysis of REG3B in cecal content (B = bacterial fraction, CW = cecal water fraction, M1–M3 = 3 individual mice of each group). (F+G) PCoA analysis of weighted UniFrac distances indicated no change in phylogenetic diversity at 3 weeks of age after cohousing (F) of WT and *Tnf^ΔARE/+^* dams and litters (n = 10–25 offspring per group) (left panel; even sampling of 28,633 sequences) or without co-housing (G) (n = 4–6 offspring per group) (right panel; even sampling of 16,865 sequences). 16S ribosomal RNA gene amplicons of the V4 region (233 bp) in caecal contents were sequenced using a MiSeq platform and analyzed as described in the methods section.

Immunofluorescence analysis of REG3B expression overtime was performed in ileal gut sections from WT and *Tnf^ΔARE/+^* offspring at 17.5 *dpc*, 3 and 8 weeks of age. In all groups, REG3B protein was not detectable in the prenatal gut, but protein expression was clearly detectable in the epithelium of 8-week-old mice (Figure 4A–C). REG3B protein expression appeared earliest in the ileal epithelium at weaning (3 weeks of age) and further increased at 8 weeks. Quantification of REG3B proteins in IEC of 8-week-old mice revealed significant reduction in iWT mice compared to WT control (*p* = 0.008), suggesting that maternal inflammation slightly affects postnatal protein expression in the absence of any tissue pathology. But, under intestinal inflammation, both ARE and iARE mice showed almost a complete loss of REG3B protein expression when compared with WT (*p*<0.0001). The loss of REG3B protein suggests that postnatal tissue inflammation profoundly impacts the expression of REG3B protein (Figure 4C and D). Tissue pathology of the distal ileum in *Tnf* ^ΔARE/+^ offspring was not detectable in the pre- and perinatal period (until 3 weeks of age), but was moderate in 8-week-old-offspring, the age where an inflammation-driven loss of epithelial REG3B occurs. With the finding that maternal and postnatal inflammation was associated with decreased REG3B expression in the epithelium, we measured REG3B in aqueous and bacterial fractions of caecal contents from 8-week-old mice in order to evaluate an alteration in luminal secreted REG3B. Representative blots revealed the presence of REG3B in bacterial fractions (Figure 4E). Large amounts of REG3B were detected in bacterial fractions of WT mice, whereas moderate and low amounts were found in WT and inflamed ARE/iARE mice, respectively.

### Caecal Bacterial Communities are not Altered in Response to Maternal Inflammation

Down-regulation of REG3B expression in healthy WT offspring suggested that maternal inflammation has a priming effect on the offspring's gut bacterial ecosystem. We were preferentially interested in early development of the microbiota due to maternal inflammation independently of postnatal inflammation and REG3B regulation. Therefore, we analyzed 16S-based diversity and composition in the caecal content from offspring at 3 weeks of age (pre-weaning). At this stage, we circumvented possible effects of postnatal diet on gut bacteria. We followed two different breeding strategies. Our first strategy was to co-house WT and *Tnf^ΔARE/+^* dams in order to synchronize the dam's microbiota prior to colonization of the pups at birth. We obtained a total of 1,755,593 quality-checked sequences (28,633 to 86,232 per sample) representing a total of 263 OTUs with an average of 122 ± 33 molecular species per sample. Members of the phyla *Firmicutes*, *Bacteroidetes* and *Proteobacteria* were dominant, with abundances of 70.9%, 21.3% and 4.6% total sequences, respectively. *Lachnospiraceae* (59.3%) and *Porphyromonadaceae* (8%) were the most abundant families. We observed no effect of maternal inflammation on the phylogenetic make-up of caecal bacterial communities between WT and iWT offspring at the age of 3 weeks, *i.e.*, there was no clustering of samples according to dam genotypes based on the analysis of weighted Unifrac distances (Figure 4F). Our second strategy was to assess whether there is a general effect of *Tnf^ΔARE/+^* dams on the offspring's microbiota compared to WT dams. Therefore, we performed a second breeding of WT and *Tnf^ΔARE/+^* dams that were housed separately. We obtained in this case a total of 1,940,009 quality-checked sequences (16,865 to 61,102 per sample) representing a total of 286 OTUs. Again, we found no influence of the maternal *Tnf^ΔARE/+^* genotype on the phylogenetic make-up of bacterial communities in caecal contents in both WT and *Tnf^ΔARE/+^* offspring (n = 4 to 6 mice per group) (Figure 4G). The number of phylotypes was also not influenced by maternal inflammation and offspring's genotype (Table S1). These data demonstrate that overall bacterial diversity is not affected by maternal inflammation or offspring's genotype before the onset of postnatal inflammation. Interestingly, analysis of the caecal microbiota at 8 weeks of age, when inflammation of the distal ileum is established (n = 4 to 6 mice per group), revealed that REG3B does not affect bacterial diversity and composition of Chow diet-fed offspring (Figure S2). Taxonomic assignment revealed no statistically significant differences in sequence abundances due to maternal or postnatal inflammation after adjustment for multiple testing (Tables S2A and S2B). However, feeding offspring a well-defined semisynthetic experimental diet revealed clear differences in bacterial diversity according to the offspring's genotype, i.e., PCoA analysis indicated a change in beta-diversity between WT and *Tnf^ΔARE/+^* offspring (n = 4–5 mice each). Statistical analysis on mean phylogenetic distances between groups (inter-group distances) clearly indicated significance (Figure S2). Besides inflammation-associated alterations driven by the offspring genotype, we did not observe any shifts in diversity in response to maternal inflammation (Fig S2D and S2F). When comparing chow-fed mice at the age of 3 and 8 weeks, we observed age-dependent shifts in *beta*-diversity independently of dam and offspring genotype (Figure S3A). Major age-dependent effects on bacterial taxa included an increase in members of the *Lactobacillaceae*, and *Prevotellaceae* as well as decreased proportions of *Lachnospiraceae* and *Deferribacteriaceae* (Figure S3B and Table S3).

## Discussion

In the present work, we provide clear experimental evidence that maternal inflammation has no impact on the offspring's risk to develop genetically-driven ileitis and colitis, despite the fact that TNF-driven maternal ileitis extensively modulates transcriptional responses in the fetal epithelium. This clearly suggests that effects of maternal inflammation are overwritten in genetically-driven models for intestinal inflammation, indicating that the disease end point might be independent of fetal exposure to inflammation[24].

CD-associated pregnancy in humans is characterized by alterations of the cytokine milieu leading to peri- and postnatal complications including preterm labor and low birth weights [6], [7]. Most importantly, we showed that 8-week-old ARE and iARE mice developed similar levels of ileal inflammation. This effect was confirmed in a second mouse model for colitis, clearly supporting the hypothesis that maternal inflammation does not affect the onset or severity of disease in the genetically susceptible offspring. Nevertheless, our experiments demonstrate for the first time that TNF-driven maternal inflammation substantially modulates the transcriptional profile in the fetal intestinal epithelium, supporting the hypothesis that not only infection-driven acute but also chronic maternal inflammation impacts on the progeny[25], [26]. We identified two clearly distinct clusters of genes that were strongly associated with fetal exposure to maternal inflammation (1,614 genes) or postnatal development of TNF-mediated tissue pathology (830 genes). In fetuses, similar patterns were observed in iWT and iARE with 1,614 common genes in both groups (around 70% overlap). The fetal ARE genotype however seemed not to be relevant at the early developmental stage (117 regulated genes), suggesting that the disease-susceptible genotype does not have a major influence on fetal gut programing. In contrast, gene expression profiles in postnatal IEC were highly influenced by the genetically-driven disease phenotype with 1,154 and 1,197 significantly regulated genes under ARE and iARE conditions, respectively. Heat map analysis confirmed the substantial overlap of similarly regulated genes (around 70%; 830 genes) in these two groups of ARE mice, implying that transcriptional fingerprints in the fetal gut were completely overwritten by signals derived from the postnatal environment independently of the disease-susceptible genotype. These findings may suggest that genetically-driven ileitis in mice is largely induced by the postnatal environment.

Gene ontology analysis identified ‘metabolic and cellular’ processes as being significantly linked to maternal inflammation to a higher extent in the fetal than in the postnatal stage. Consistent with the over representation of ‘metabolic processes’, *Fabp6* was strongly up-regulated in the fetal gut in response to maternal inflammation. FABP6 facilitates efficient transepithelial transport of both bile and fatty acids [27]. Although other biological processes such as ‘localization’ and ‘death’ were not influenced to the same extent as ‘metabolic and cellular processes', they included top-regulated genes such as *C3*, *S100a8* and *Lyz2* in both ARE and iARE offspring. This reflects a disease-associated inflammatory program[28], [29] as well as bacterial defense mechanisms[30], [31]. Additionally, the strong down-regulation of *Il18* by approximately 25-fold, hints at the relevance of missing immune tolerance in the *Tnf^ΔARE/+^* model for CD[32].

Both the fetal and postnatal stage revealed a significant changes within the GO category ‘response to stimulus’, albeit with lower number of observed genes when compared with top-categories like ‘metabolic and cellular processes'. Interestingly, this category comprises *Reg3b* as top-regulated gene under maternal inflammation at 17.5dpc. The *Reg*3 gene family belongs to a group of C-type lectins, based on their carbohydrate recognition domains[33]. Interestingly, REG3B protein was not detectable in the prenatal gut, but was clearly visible in the epithelium of mice at 8 weeks of age. Immunofluorescence analysis of REG3B expression over time showed appearance earliest at weaning (3 weeks). This observation is consistent with a previously published *Reg3g* expression analysis from Matsumoto *et al*.[34]. The closely related lectin REG3G is known to drive host-bacterial segregation[35], preferentially targeting Gram-positive bacteria[36]. Thus, loss of REG3B seems to be associated with inflammatory signals at postnatal stages and may contribute to the development of genetically-driven ileitis through mechanisms that involve the intestinal microbiota of *Tnf* ^ΔARE/+^ mice[16], [37]. Quantification of REG3B protein levels in IEC of 8-week-old mice revealed significant reduction in iWT, suggesting that maternal inflammation can slightly affect postnatal protein expression. However, ARE and iARE mice almost completely lost REG3B, suggesting that postnatal tissue inflammation profoundly impacts REG3B expression independently of earlier gene transcriptional up-regulation by maternal inflammation. This is supported by the fact that, fetal epithelial programming is completely overwritten in adult offspring, clearly suggesting that postnatal disease-relevant signals initialize genetically-driven ileal pathogenesis.

Interestingly, loss of REG3B in the epithelium was linked to lower REG3B levels preferably bound to bacterial fractions in the caecal content, clearly suggests that the postnatal microbiota harbors disease-relevant signals that show stronger potential to initialize the genetically-driven ileal pathogenesis compared to maternal stimuli. This is supported by the fact that maternally-induced changes in the fetal epithelium did not cause any shifts in diversity and composition of caecal bacterial communities at 3 weeks of age before weaning. This was shown in two different breeding strategies, with and without co-housing of WT and *Tnf^ΔARE/+^* dams. However, at 8 weeks of age, we observed an inflammation-driven loss of REG3B in IEC and bacterial fractions of the caecal content that is not additionally affected by maternal inflammation. The fact that the microbial composition and diversity was not affected by maternal or offspring's inflammation indicates that REG3B expression in the epithelium has no influence on the overall phylogenetic make-up of caecal bacterial communities in the *Tnf^ΔARE/+^* mouse model. We cannot fully explain this absence of any major signs of maternal or inflammation-driven dysbiosis in the offspring's microbiota despite changes in REG3B expression. Diet might be one main confounding factor, since wheat-based Ssniff Chow diets are characterized by highly varying quality due to heterogeneity of raw products used during production. This suggestion was supported by clear inflammation-driven shifts in *beta*-diversity between WT and ARE mice when fed a well-defined semisynthetic experimental diet based on corn starch.

Taking all these findings together, we summarize that neither maternal nor genetically-driven inflammation impact the overall phylogenetic diversity of caecal bacterial communities in the offspring under conventional conditions. The observation of age-dependent shifts in bacterial diversity and composition between 3 and 8 weeks of age goes in line with a previously published work by Garrett et al. [12]. But, unlike their findings that a colitogenic microbiota is transmitted from mothers to offspring under SPF conditions, we could not observe any changes through maternal inflammation in our experiments. Altogether, we conclude from our studies that maternal inflammation impacts the ileal transcriptome in fetuses, but these effects do not persist in grownup mice and are therefore not relevant for the modulation of intestinal inflammation in the genetically susceptible *Tnf^ΔARE/+^* mouse. Consistent with these findings is the occurrence of age- and diet- but not maternal-dependent shifts in bacterial diversity and composition, indicating that postnatal factors largely overwrite a possible maternal influence on the offspring's microbial ecosystem in both non-disease and disease susceptible offspring. This is also in line with the fact that the host immune response at the site of inflammation is consistently unaffected by maternal inflammation in two different mouse models for genetically-driven ileitis and colitis inflammation. Consequently, maternal inflammation during gestation in mouse models did not alter the genetically-driven risk to develop chronic inflammation in the intestine.

## Supporting Information

Figure S1 Fetal environment and laser microdissection of fetal intestinal epithelial cells. (**A**) Randomly selected fetuses of the last 5 Theiler stages (TS) (15–19 *dpc*). (**B**) Macroscopic view of a 17.5 *dpc* gut and subsequent laser microdissection procedure of fetal ileal epithelium. Epithelial areas of 1.33 ± 0.024×10^6^ µm^2^ (mean ± SD) were cut for microarray analysis.
(TIF)

Figure S2 Experimental diet clearly influences changes in caecal bacterial diversity in response to postnatal but not maternal inflammation. Histological scores of terminal ileum from WT, iWT, ARE and iARE offspring on (**A**) chow diet (experiment from Figure 2A, n = 5–7 mice each), or (**B**) experimental diet (n = 6–16 mice each). With both diets, there was no difference in inflammatory scores relative to maternal inflammation. (**C+E**) Analysis of phylogenetic distances indicated no significant change in *beta*-diversity between offspring fed the Ssniff chow diet. Comparisons of mean phylogenetic distances (weighted UniFrac) between individual mice (WT, iWT, ARE, iARE) within groups (intra-group distances, e.g. all WT) and between mice from different groups (inter-group distances, e.g. WT vs. iWT) revealed no significant differences related to the offspring's genotype or maternal inflammation. (**D+F**) PCoA analysis indicated an inflammation-driven change in *beta*-diversity between 8-week-old WT and *Tnf^ΔARE/+^* offspring fed an experimental diet (n = 4–5 mice each). Statistical comparisons of phylogenetic distances indicated significant separation between WT and ARE or iWT and iARE but not between WT and iWT or ARE and iARE (Two-Way ANOVA, ***p>0.0001).
(TIF)

Figure S3 Age-dependent shifts in bacterial diversity and composition. (**A**) PCoA analysis indicated a change in diversity between mice at the age of 3 and 8 weeks (n = 18–19). (**B**) Major bacterial taxa that were characterized by significantly different sequence proportions at 3 and 8 weeks of age are shown in box plots (F-test followed by Benjamini-Hochberg adjustment). Individual data for all taxa are given in Table S3.
(TIF)

Table S1 Observed phylotype numbers in caecal contents of WT, iWT, ARE and iARE offspring at 3 and 8 weeks of age. Indicated are means ±SD (n = 4–5 per group). Three Way ANOVA.
(XLSX)

Table S2 Sequence proportions of bacterial taxa in ceacal contents from offspring (n = 4–5) at (A) 3 or (B) 8 weeks of age. OTUs occurring in less than 2 mice and <0.05% total sequences per sample were excluded from the analysis. Sequence proportions were analyzed for significant differences using F-Test followed by Benjamini-Hochberg correction for multiple testing in the R programing environment after adjustment for multiple testing.
(XLSX)

Table S3 Sequence proportions of bacterial taxa in ceacal contents from 3- and 8-week old offspring (n = 4–6) show age differences. OTUs occurring in less than 2 mice and <0.05% total sequences per sample were excluded from the analysis. Sequence proportions were analyzed for significant age-differences using F-Test followed by Benjamini-Hochberg correction for multiple testing in the R programing environment after adjustment for multiple testing.
(XLSX)
