# Supplementary figures and images for: Fetal Exposure to Maternal Inflammation Does Not Affect Postnatal Development of Genetically-Driven Ileitis and Colitis
Source: PLoS One. 2014 May 21;9(5):e98237. doi: 10.1371/journal.pone.0098237 (PMC4029898; doi:10.1371/journal.pone.0098237)

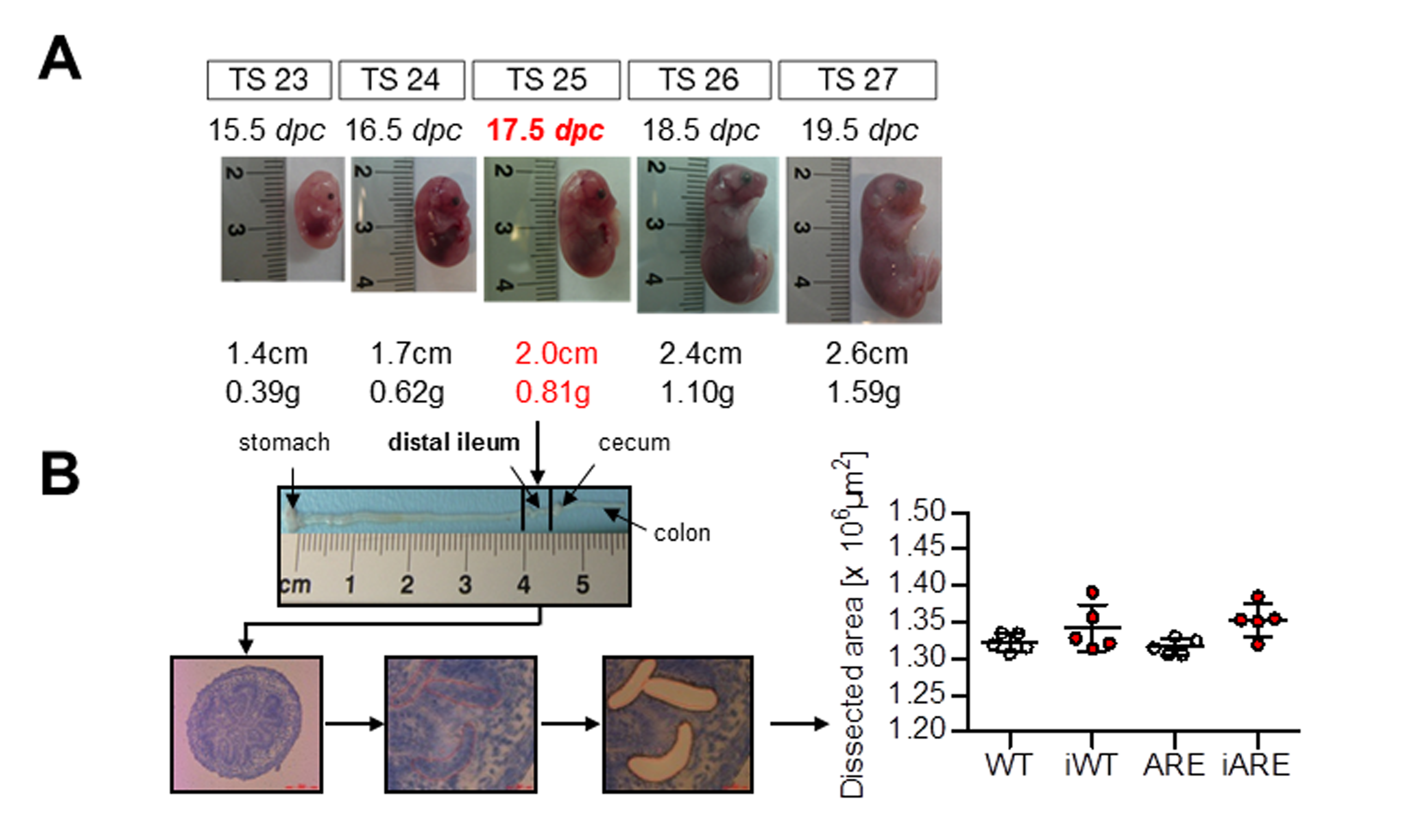

Supplement: Figure S1 — Fetal environment and laser microdissection of fetal intestinal epithelial cells. (A) Randomly selected fetuses of the last 5 Theiler stages (TS) (15–19 dpc). (B) Macroscopic view of a 17.5 dpc gut and subsequent laser microdissection procedure of fetal ileal epithelium. Epithelial areas of 1.33 ± 0.024×10^6^ µm2 (mean ± SD) were cut for microarray analysis. (TIF) [file pone.0098237.s001.tif]

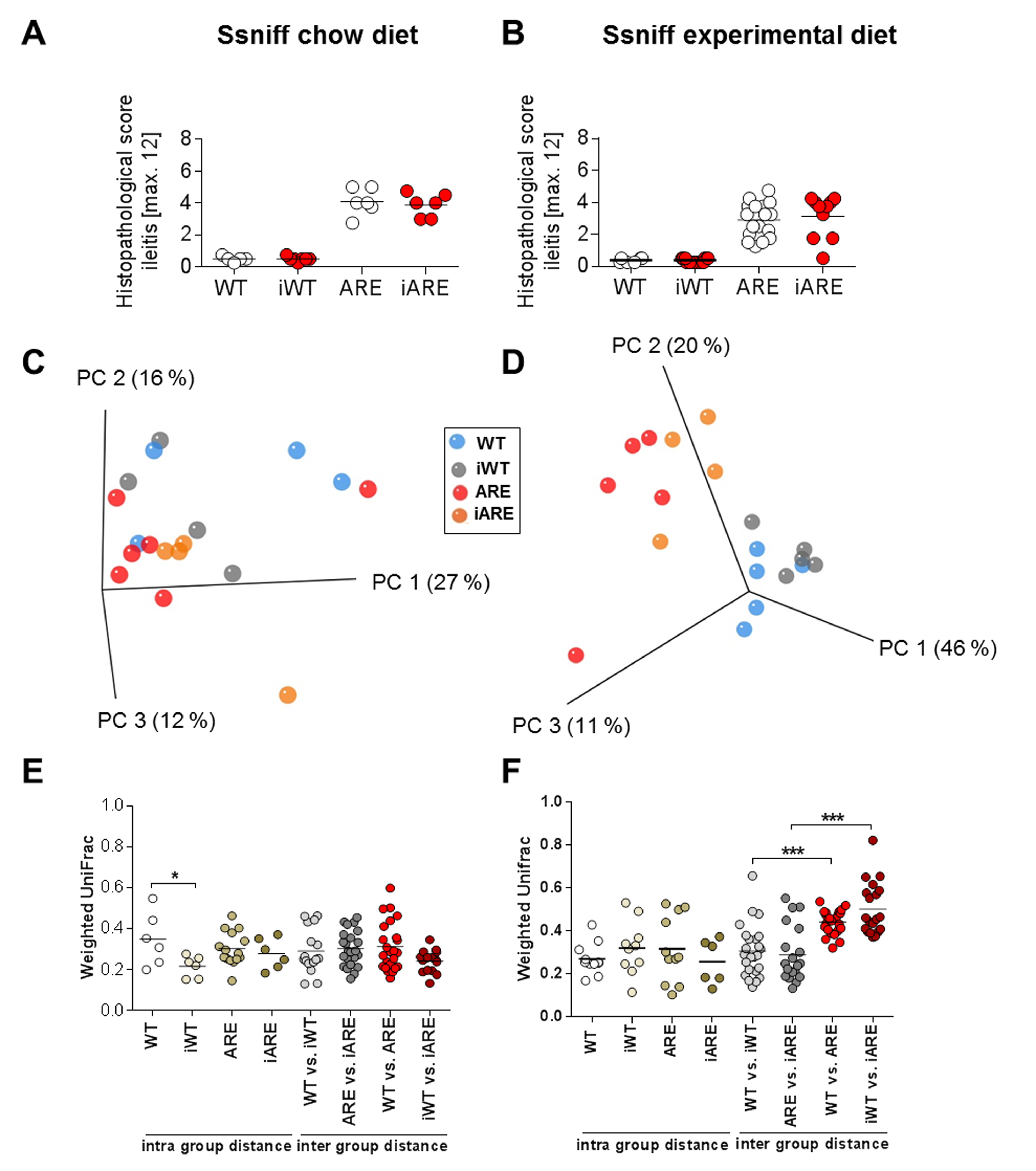

Supplement: Figure S2 — Experimental diet clearly influences changes in caecal bacterial diversity in response to postnatal but not maternal inflammation. Histological scores of terminal ileum from WT, iWT, ARE and iARE offspring on (A) chow diet (experiment from Figure 2A, n = 5–7 mice each), or (B) experimental diet (n = 6–16 mice each). With both diets, there was no difference in inflammatory scores relative to maternal inflammation. (C+E) Analysis of phylogenetic distances indicated no significant change in beta-diversity between offspring fed the Ssniff chow diet. Comparisons of mean phylogenetic distances (weighted UniFrac) between individual mice (WT, iWT, ARE, iARE) within groups (intra-group distances, e.g. all WT) and between mice from different groups (inter-group distances, e.g. WT vs. iWT) revealed no significant differences related to the offspring's genotype or maternal inflammation. (D+F) PCoA analysis indicated an inflammation-driven change in beta-diversity between 8-week-old WT and TnfΔARE/+ offspring fed an experimental diet (n = 4–5 mice each). Statistical comparisons of phylogenetic distances indicated significant separation between WT and ARE or iWT and iARE but not between WT and iWT or ARE and iARE (Two-Way ANOVA, ***p>0.0001). (TIF) [file pone.0098237.s002.tif]

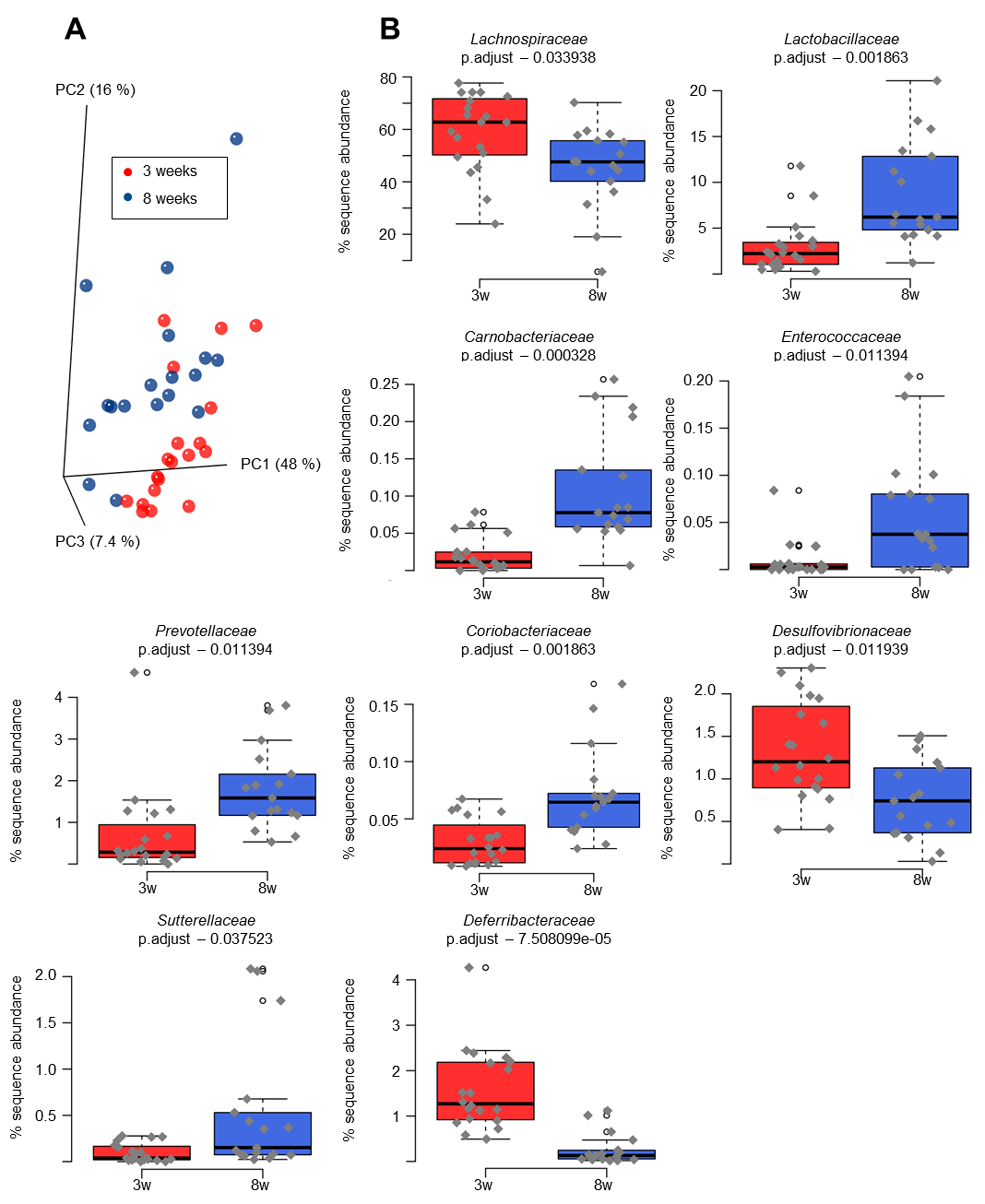

Supplement: Figure S3 — Age-dependent shifts in bacterial diversity and composition. (A) PCoA analysis indicated a change in diversity between mice at the age of 3 and 8 weeks (n = 18–19). (B) Major bacterial taxa that were characterized by significantly different sequence proportions at 3 and 8 weeks of age are shown in box plots (F-test followed by Benjamini-Hochberg adjustment). Individual data for all taxa are given in Table S3. (TIF) [file pone.0098237.s003.tif]
